# The preventable efficacy of β-glucan against leptospirosis

**DOI:** 10.1371/journal.pntd.0007789

**Published:** 2019-11-01

**Authors:** Jiaqi Wang, Zhao Jin, Wenlong Zhang, Xufeng Xie, Ning Song, Tianbao Lv, Dianjun Wu, Yongguo Cao

**Affiliations:** 1 Department of Clinical Veterinary Medicine, College of Veterinary Medicine, Jilin University, Changchun, People’s Republic of China; 2 Key Laboratory for Zoonosis Research, Ministry of Education, College of Veterinary Medicine, Jilin University, Changchun, People’s Republic of China; Instituto Butantan, BRAZIL

## Abstract

Leptospirosis, caused by pathogenic *Leptospira* species, has emerged as an important neglected zoonotic disease. Few studies have reported the preventable effects of immunoregulators, except for antibiotics, against leptospirosis. Generally, immunostimulatory agents are considered effective for enhancing innate immune responses. Many studies have found that beta-glucan (β-glucan) could be a potent and valuable immunostimulant for improving immune responses and controlling diseases. In this study, we investigated the preventable role of β-glucan against *Leptospira* infection in hamsters. First, β-glucan was administered 24 h prior to, during and after infection. The results showed that β-glucan increased the survival rate to 100%, alleviated tissue injury, and decreased leptospire loads in target organs. Additionally, we found using quantitative real-time PCR that application of β-glucan significantly enhanced the expression of Toll-like receptor (TLR) 2, interleukin (IL)-1β and iNOS at 2 dpi (days post infection) and reduced the increase of TLR2, IL-1β and iNOS induced by *Leptospira* at 5 dpi. Furthermore, to induce memory immunity, β-glucan was administered 5 days prior to infection. β-Glucan also significantly increased the survival rates and ameliorated pathological damage to organs. Moreover, we demonstrated that β-glucan-trained macrophages exhibited elevated expression of proinflammatory cytokines (IL-1β and IL-6) *in vitro*, indicating that β-glucan induces an enhanced inflammatory response against *Leptospira* infection. These results indicate that administration of β-glucan and other immunostimulants could be potential valuable options for the control of *Leptospira* infection.

## Author summary

Leptospirosis, an important emerging neglected zoonotic disease, is caused by *Leptospira* and affects humans as well as animals. Due to the emergence of bacterial resistance to antibiotics, the development of alternatives to antibiotics has become an inevitable requirement in this new situation. Immunoregulators act as biological response regulators that do not induce toxicity, side effects, or resistance and can enhance, regulate, and restore nonspecific immunity to a host's immune response. β-Glucan, an immunostimulant, increased the survival rate, alleviated tissue injury, and decreased the abundance of leptospires in target organs. β-Glucan enhanced the inflammatory response, which was associated with enhanced prevention hamsters from *Leptospira* infection. Our findings also demonstrated that β-glucan-induced trained immunity protected against *Leptospira* infection. These results contributed to an explanation for the preventable mechanism against *Leptospira* infection and revealed that β-glucan and even other immunostimulants could be potent and valuable agents for controlling *Leptospira* infection.

## Introduction

Leptospirosis, an important emerging neglected zoonotic disease, is caused by *Leptospira* and affects humans as well as animals. Its clinical severity ranges from asymptomatic to life-threatening disease characterized by hepatorenal failure with or without pulmonary hemorrhage. Although rodents are frequently the source of bacteria causing human infections, many mammalian species have been found to harbor infection in their kidneys [1–4]. Antibiotic therapy is effective in the management of patients with leptospirosis [2]; however, antibiotics cause toxicity and side effects, moreover drug resistance and double infections can occur [5]. Therefore, the development of alternatives to antibiotics has become an inevitable requirement in this new situation. Immunoregulators acts as biological response regulators that do not induce toxicity, side effects, or resistance and can enhance, regulate, and restore nonspecific immunity to the body's immune response [6]. However, less is known about the protective and preventable effect of immunoregulators against leptospirosis.

It has been widely reported that immunosuppressive agents protect the host from *Leptospira* infection [7,8], whereas in previous studies, it was found that the inflammatory storm in the susceptible model after infection was delayed and more severe than in the tolerant model [9]. The innate immune system constitutes the first line of host defense, playing a crucial role in the early recognition and elimination of leptospires [10]. Thus, we speculated that the application of immunostimulants plays a role in preventing leptospirosis by activating innate immunity. Beta-glucans (β-glucans) are glucose polymers found in the wall of yeast cells and bacteria as well. In mammals, β-glucans have been shown to activate innate immunity through Toll-like receptor (TLR) 2/TLR6 heterodimers [11] and though recognition by the small membrane receptor dectin-1 [12]. Therefore, we investigated whether β-glucan, as an immunostimulant, prevents *Leptospira* infection by activating the inflammatory response.

The ability of the innate immune system to respond adaptively to infections is a genuine characteristic of innate immune memory—a process known as "training immunity" [13]—the impact of which on our understanding of innate immune responses has become increasingly obvious. In recent years, the molecular mechanisms that lead to trained immunity in mammalian cells have been described. These studies have focused mainly on natural killer cells [14–16] and monocytes [17–19]. Macrophages derived from these β-glucan-primed monocytes exhibit a degree of training or memory and respond with an increased release of inflammatory cytokines to subsequent infections with related or unrelated pathogens [17–22]. However, the mechanism of β-glucan-primed trained immunity against *Leptospira* infection is unclear.

TLRs acting as pattern recognition receptors (PRRs) can recognize a variety of pathogen-associated molecular patterns (PAMPs) [9]. Many studies have shown that TLRs, particularly TLR2, play a crucial role in the development of leptospirosis [7,23]. TLR Expression of TLRs results in the induction of inflammatory cytokine expression. The inflammatory response is a common feature in all patients with leptospirosis, although the clinical signs differ among patients [3,24]. Inflammation, which is mediated by proinflammatory factors produced by the host, is an immunoprotective response during the early stages of infection [6]. However, excessive production of proinflammatory cytokines also causes pathological inflammatory disorders and tissue injury [25]. IL-1β, IL-6 and IL-10 were shown to be involved in inflammatory responses during *Leptospira* infection [24,26]. Moreover, nitric oxide (NO) production is a potent innate mechanism to eliminate invading bacteria. It has been previously reported that *Leptospira* induce the upregulation of iNOS mRNA in kidneys at 3 dpi. [10]. In that study, the researchers confirmed NO production upon stimulation of bone marrow macrophages with live or dead *Leptospira*. However, NO that is excessively formed by iNOS intensifies the inflammatory reaction and causes damage to tissues [27,28]. β-Glucan affects the release of NO by macrophages [26,29].

In the present study, we investigated the effect of β-glucan against leptospirosis. First, we showed the ability of β-glucan to prevent hamsters from leptospirosis *in vivo*. Our results showed that hamsters administered β-glucan prior to *Leptospira* infection displayed an increased inflammatory response in advance of challenge, which was associated with enhanced protection against *Leptospira* infection. We also demonstrated that β-glucan-induced trained immunity protected against *Leptospira* infection *in vitro*. These results contributed to an explanation of the prevention mechanism against *Leptospira* infection and revealed that β-glucan and even other immunostimulants could be potent and valuable agents for controlling *Leptospira* infection.

## Materials and methods

### Ethics statement

All animals were maintained on standard rodent chow with water supplied *ad libitum* under a 12 h/12 h light/dark cycle during the experimental period. All animal experiments followed the regulations on animal welfare and Public Health Service recommendations in China. The protocol was approved by the Committee on the Ethics of Animal Experiments of the First Norman Bethune Hospital of Jilin University, China [(2013) clinical trial (2013–121)].

### Bacterial strains and growth conditions

*Leptospira interrogans* serovar Lai (56601) was kindly provided by Xiaokui Guo. The strain was cultivated at 29°C in Ellinghausen-McCullough-Johnson-Harris (EMJH) liquid medium, and pathogenicity was maintained by passage in hamsters[30]. The concentration of bacterial cells was determined by using a Petroff-Hausser counting chamber and dark-field microscopy. The strain had undergone <5 *in vitro* passages before being used to infect hamsters.

### Effect of β-glucan on *Leptospira* growth

To analyze the influence of β-glucan (Solarbio, China) on *Leptospira* growth, 10^7^ leptospires in 1 ml of 0.9% saline supplemented with or without 5 mg/10 mg of β-glucan, which was dissolved in sterile water were cultured for 1 h, at 29°C. Growth was analyzed for 4 days by using a Petroff-Hausser chamber and a dark-field microscope.

### *In vivo* experiments

Leptospirosis models in hamsters were established as previously reported [9]. The 7-day mortality rate was 100% after challenge with *Leptospira* [7]. All golden Syrian hamsters were challenged by intraperitoneal injection with 1 ml of 0.9% NaCl containing 10 ^7^ leptospires of strain 56601, which were counted using a Petroff–Hausser counting chamber under dark-field microscope.

Early prevention experiments: One day before infection, experimental group hamsters were intraperitoneally injected with β-glucan (5mg/kg, 10mg/kg) resuspended in 0.9% saline, and control hamsters were intraperitoneally injected with 0.9% normal saline. At the time of infection, each hamster was intraperitoneally injected with 1 ml of 0.9% saline (all lethal doses) containing 10^7^ *Leptospira*; the experimental group was injected with drugs, and the control group with physiological saline. One day after infection, the experimental group and the control group were injected with drugs or saline, respectively. In addition, a similar treatment was added that the experimental group was injected with β-glucan(10mg/kg) and the Histopathological Examination and the *Leptospira* burdens in organs were performed on the fourth day after infected because the infected control group began to die.

Trained immunization experiment: Five days before infection, experimental group hamsters were intraperitoneally injected with β-glucan (10 mg/kg) in 0.9% normal saline, and control hamsters were intraperitoneally injected with 0.9% normal saline. At the time of infection, each hamster was intraperitoneally injected with 1 ml of 0.9% saline (all lethal doses) containing approximately 10^7^ *Leptospira*.

In the hamster *Leptospira* infection model, sampling and testing was conducted during the 21-day experimental period. Surviving hamsters were humanely euthanized after 21 days using CO_2_.

### *In vitro* experiments

Culture of primary hamster macrophages: Hamsters were injected with 2 ml of 3% thioglycolate medium (BD Biosciences, Sparks, MD). Three days after the injection, peritoneal macrophages were isolated by washing the peritoneal cavity with dulbecco's modified eagle medium (DMEM, HyClone^TM^). Cells were cultured at 37°C in 5% CO_2_ in DMEM supplemented with 10% fetal bovine serum. A total of 1X10^6^ cells in each well were seeded in 6-well culture plates in 2 ml of fresh culture medium. After incubation for 6 hours at 37°C and 5% CO_2_, each experimental well was prestimulated for 24 hours with 0.5 ml of β-glucan (10 mg/ml) suspended in DMEM. After 5 days, each well was stimulated with *Leptospira* at an MOI of 100 per cell. Cells were then incubated for 24 h; thereafter, cells were harvested, and total RNA extraction was performed.

### Histopathological examination

Animals were humanely euthanized with CO_2_, and primary organs (liver, kidneys, and lungs) were immediately removed. Organs for histopathological examination were fixed in 10% formalin for 48–72 h, dehydrated in a gradient alcohol series, embedded in paraffin, and stained with hematoxylin and eosin (H&E).

The severity of leptospire-induced lesions was graded as described previously [31]. Briefly, tubulointerstitial nephritis was graded as follows: 0 for normal, 1 for mild, 2 for moderate, and 3 for severe. Liver pathology was graded based on the average number of inflammatory foci in 10-by-10 fields, as follows: 0 for normal, 1 for 1 to 3 foci, 2 for 4 to 7 foci, and 3 for 7 foci. The extent of pulmonary hemorrhage was graded as follows: 0 for no hemorrhage, 1 for a single focus, 2 for multiple foci, and 3 for locally extensive hemorrhage.

### DNA extraction and quantitative PCR (qPCR)

The *Leptospiral* burdens in infected organs were determined by quantitative PCR (qPCR) using an Applied Bioscience 7500 thermocycler and FastStart Universal SYBR Green Master (Roche Applied Science, Germany). Tissue DNA was extracted using a TIANamp Bacteria DNA kit (Tiangen, China) according to the manufacturer’s instructions. The qPCR primer pair used was LipL32-f (5’-CGCTTGTGGTGCTTTCGGTG-3’) and LipL32-r (5’-GCGCTTGTCCTGGCTTTACG-3’). The resulting amplicon was 152 bp[32,33]. The PCR protocol consisted of an initial incubation step at 95°C for 12.5 min followed by 40 cycles of amplification (95°C for 15 s, 62°C for 30 s and 72°C for 30 s). The concentration of DNA was measured by spectrophotometry. The genomic DNA from a counted number of leptospires was used as a calibrator for qPCR. The results are expressed as the number of genome equivalents per milligram of organ DNA.

### Real-time quantitative PCR

Cells or 0.05 to 0.1 g of organ tissues were homogenized in TRIzol reagent, and total RNA was extracted according to the TRIzol protocol (Invitrogen, USA). RNA was reverse transcribed to cDNA by using random primers from a TransScript One-Step gDNA Removal Kit and cDNA Synthesis SuperMix (TransGen Biotech, China). Quantification of relative mRNA concentrations was conducted by using an Applied Bioscience 7500 thermocycler and FastStart Universal SYBR Green Master (Roche Applied Science, Germany). The PCR conditions were as follows: 50°C for 2 min and 95°C for 10 min, followed by 45 cycles of amplification at 95°C for 15 s and 60°C for 60 s. The primers selected for this study are listed in Table 1. By using the EQUATION method, the expression level of the target gene was normalized to the expression level of the housekeeping gene glyceraldehyde-3-phosphate dehydrogenase (GAPDH). Every sample was run in triplicate.

**Table 1:** Sequence of primers used for qPCR assays.

Gene	Primer	Sequence (5f pri
Hamster GAPDH	Sense	GATGCTGGTGCCGAGTATGT
	Anti-sense	GCAGAAGGTGCGGAGATGA
Hamster TLR2	Sense	TGTTTCCCGTGTTACTGGTCAT
	Anti-sense	CACCTGCTTCCAGACTCACC
Hamster TLR4	Sense	ACGACGAGGACTGGGTGAGA
	Anti-sense	GCCTTCCTGGATGATGTTGG
Hamster IL-6	Sense	AAGTCGGAGGTTTGGTTACAT
	Anti-sense	GGAGGCACTCATTTATT
Hamster iNOS	Sense	GGAGCGAGTTGTGGATTGTC
	Anti-sense	CCTGGGAGGAACTGATGGA
Hamster IL-10	Sense	AAGGGTTACTTGGGTTGCC
	Anti-sense	AATGCTCCTTGATTTCTGGC
Hamster IL-1β	Sense	TTCTGTGACTCCTGGGATGGT
	Anti-sense	GTTGGTTTATGTTCTGTCCGTTG

### Data analysis

SPSS version 19.0 was used to create Kaplan-Meier plots for all experiments. Survival differences between the study groups were evaluated for statistical significance using a two-sided paired t-test by SPSS software. A difference was considered significant if the P value was <0.05.

## Results

### β-glucan prevent hamsters from *Leptospira* infection

**Fig 1:**
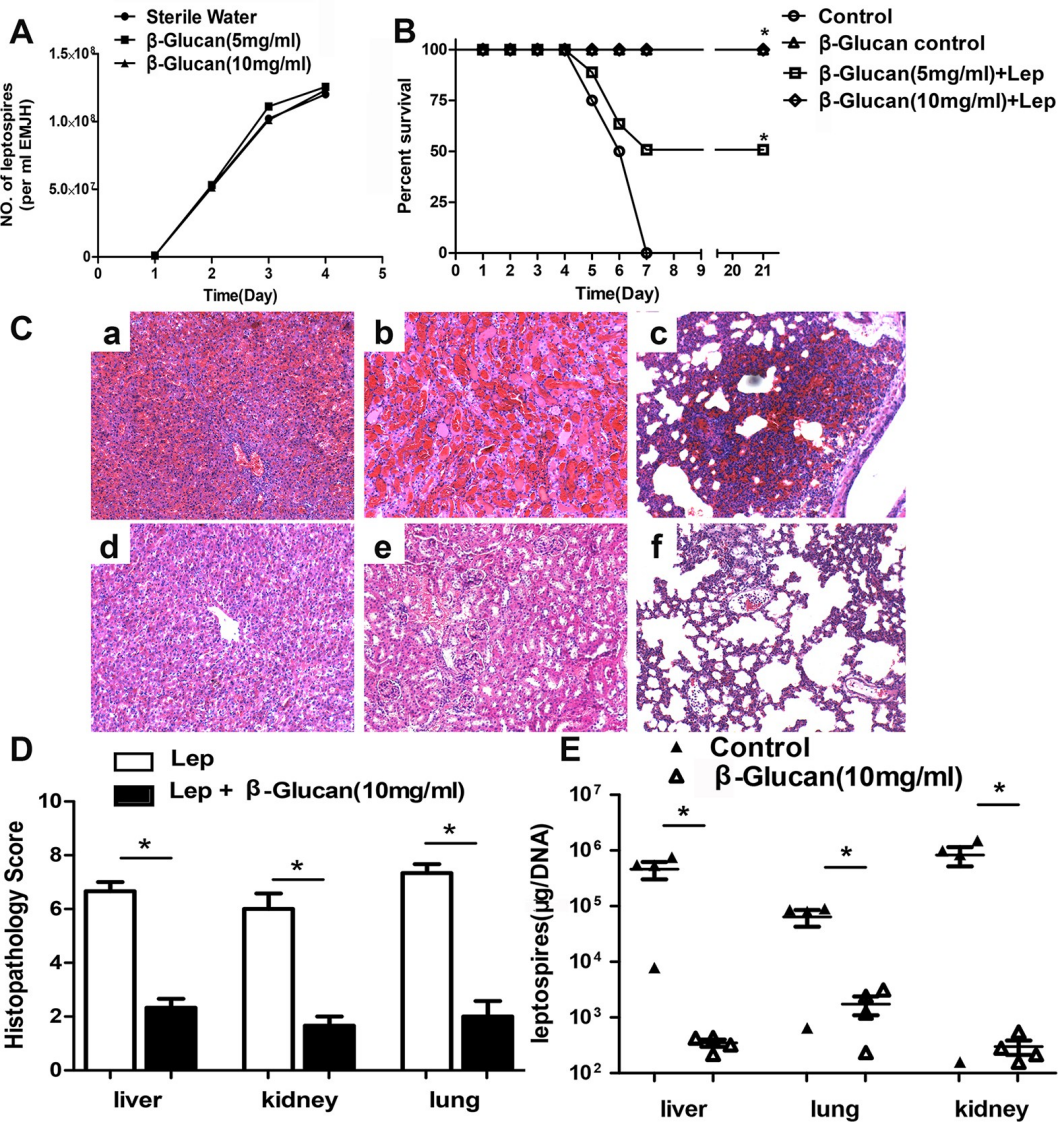
Influence of β-glucan on the pathology of hamsters with leptospirosis. (A) Effect of β-glucan on Leptospira growth. A total of 10^7^ leptospires in 1 ml of 0.9% saline supplemented with or without 5 mg or 10 mg of β-glucan were cultured at 29°C. Growth was analyzed for 4 days by using a Petroff-Hausser chamber and a dark-field microscope. Each data point represents the means ± standard deviations from three independent experiments. P values of < 0.05 were considered significant (*, P < 0.05). (B) Survival curves of hamsters in the infected control group (n = 8), the β-glucan control group (n = 8) and the group coinjected with leptospires and β-glucan (n = 8). Hamsters were stimulated with β-glucan 1 day prior to *leptospira* challenge and on day 0 and day 1 during *leptospira* challenge. *, P < 0.05 versus untreated controls, as determined by Kaplan-Meier analysis with a log-rank test. (C) Histopathology of the kidneys (a and b), livers (c and d), and lungs (e and f) of hamsters in the infected control group (a, c, and e) and the group coinjected with leptospires and β-glucan (b, d, and f). Magnification, 100×. Samples were collected on the 4^th^ day after infected *leptospira*, and representative images are shown. (D) Histopathology scores for kidneys, livers, and lungs of hamsters. The data are presented as the mean histopathology scores for the two groups of hamsters. Statistical analysis of the results for the infected control group (n = 4) and the group coinjected with leptospires and β-glucan was performed by using the Wilcoxon rank sum test. *, P < 0.05. Magnification, × 200. (E) Leptospiral burdens in the kidneys, livers, and lungs of hamsters in the infected control group and the group coinjected with leptospires and β-glucan, as determined by qPCR. Samples were collected on the 4^th^ day after infected *leptospira*. The results are presented as the number of genomic equivalents per microgram of tissue DNA, and the differences were compared by one-way ANOVA. *, P < 0.05.

To test the role of β-glucan against leptospirosis in hamsters, β-glucan was administered prior to, during and after *Leptospira* infection. We analyzed the effect of β-glucan on *Leptospira* growth. The data revealed that β-glucan did not affect *Leptospira* growth over a 4-day period (Fig 1A).

Hamsters challenged with *Leptospira* began to die on the fourth day after challenge, and the 21-day survival rate was 0%. Death in the low-concentration β-glucan group infected with *Leptospira* was delayed, and the 21-day survival rate was increased to 50%. Surprisingly, the application of a high concentration of β-glucan improved the survival rate of mice infected with *Leptospira* to 100%. There were no deaths in the β-glucan control group (Fig 1B).

The liver, kidney and lung lesion grades were lower in the hamsters coinjected with β-glucan and leptospires than in the infected controls (Fig 1D). Representative images of hamster livers, kidneys and lungs were selected from the group coinjected with β-glucan and leptospires and from the infected controls. The livers of infected control hamsters showed more inflammatory foci and a wider intercellular space than did those of hamsters coinjected with β-glucan and leptospires (Fig 1Ca and 1Cb). Dramatic lesions with hemorrhage were found in renal tissues of the infected controls (Fig 1Cc). In contrast, the kidneys of hamsters coinjected with β-glucan and leptospires showed some evidence of hemorrhage (Fig 1Cd). Severe pulmonary hemorrhages were found in the lungs of infected controls, whereas few hemorrhagic foci were found in hamsters coinjected with β-glucan and leptospires (Fig 1Ce and 1Cf).

The *Leptospira* burdens in the kidneys, liver, and lungs of hamsters from the group coinjected with β-glucan and *Leptospires*, and from those in the infected control group were measured by qPCR. A lower *Leptospira* burden in organs was found in the hamsters coinjected with β-glucan and leptospires than in the infected controls. (Fig 1E)

### β-glucan causes the inflammatory storm to arrive early at 2 dpi after the challenge and decrease at 5 dpi

**Fig 2:**
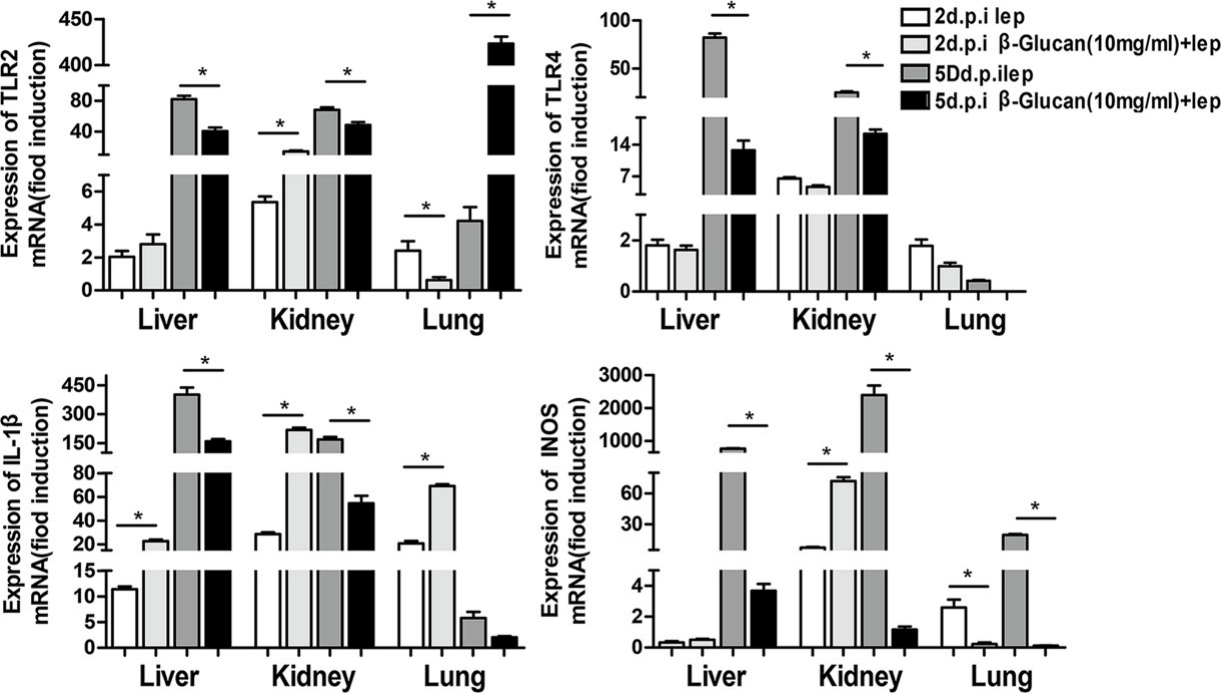
Modulation of TLR2, TLR4, IL-1β and iNOS mRNA expression in tissues after injection of leptospires. The TLR2, TLR4, IL-1β and iNOS mRNA levels in the kidneys, livers, and lungs of hamsters at 2 dpi and 5 dpi were quantified by RT-qPCR. The results were normalized to the expression level of the housekeeping gene GAPDH. The bars show the levels of TLR2, TLR4, IL-1β and iNOS (means ± standard deviations) in each tissue of hamsters (n = 3). The levels of TLR2, TLR4, IL-1β and iNOS in different tissues from three healthy individuals at 0 h were assigned a value of 1.0. Different mRNA expression levels between the infected group and the healthy group (0 h) were compared by one-way ANOVA. *, P < 0.05.

The mRNA expression of TLR2 in the liver and kidneys was significantly higher at 2 dpi and lower at 5 dpi in the group coinjected with β-glucan and leptospires than in the infected controls. However, TLR2 mRNA expression in the lungs was significantly decreased in the group coinjected with β-glucan and leptospires (Fig 2). The mRNA expression of TLR4 was reduced or induced at a lower level at 2 dpi and was clearly reduced at 5 dpi in the group coinjected with β-glucan and *Leptospires*. The mRNA expression of IL-1β in all organs was higher at 2 dpi but lower at 5 dpi in the group coinjected with β-glucan and leptospires than in the infected controls. These results indicated that β-glucan caused massive mRNA expression of TLR2 and IL-1β in organs at 2 dpi and that the expression of TLR2, TLR4 and IL-1β was reduced at 5 dpi.

### β-glucan-induced trained immunity prevent hamster from *Leptospira* infection

**Fig 3:**
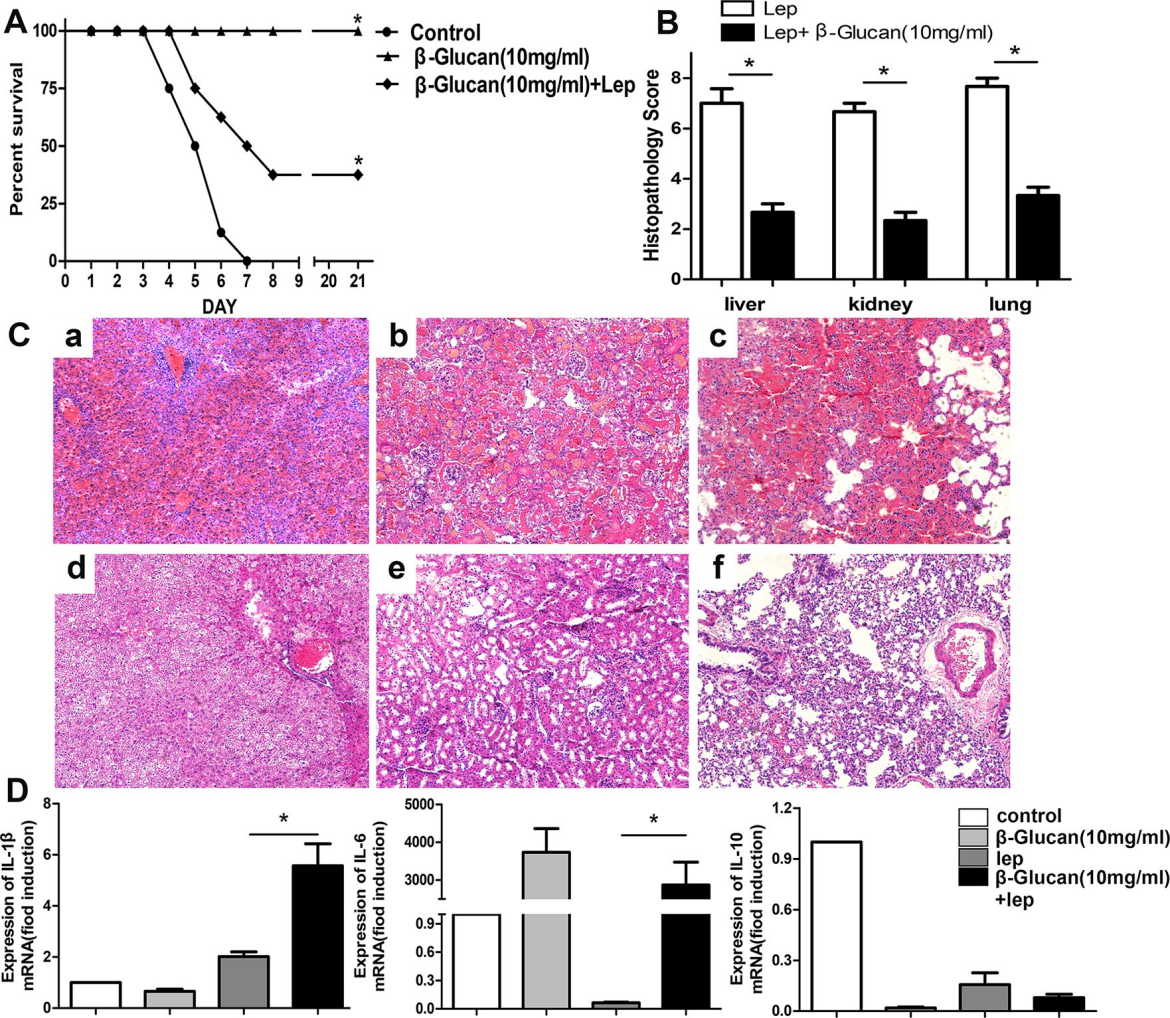
Effect of β-glucan-induced trained immunity on leptospirosis. (A) Survival curves of hamsters in the infected control group (n = 8), the β-glucan control group (n = 8) and the group stimulated with β-glucan and leptospires (n = 8). Hamsters were stimulated with β-glucan 5 days prior to *leptospira* challenge. *, P < 0.05 versus untreated controls, as determined by Kaplan-Meier analysis with a log-rank test. (B) Histopathology scores for the kidneys, livers, and lungs of hamsters. The data are presented as the mean histopathology scores for the two groups of hamsters. Statistical analysis of the results for the infected control group (n = 8) and the group stimulated with leptospires and β-glucan (n = 8) was performed by using the Wilcoxon rank sum test. *, P < 0.05. (C) Histopathology of the kidneys (a and b), livers (c and d), and lungs (e and f) of hamsters in the infected control group (a, c, and e), and the group stimulated with leptospires and β-glucan (b, d, and f). Magnification, 100×. Samples were collected over the 21-day experimental period, and representative images are shown. (D) The expression of proinflammatory factors in hamster peritoneal macrophages trained by β-glucan. Hamster peritoneal macrophages were stimulated with β-glucan 5 days prior to *leptospira* challenge. The levels of IL-1β, IL-6 and IL-10 at 1 dpi were measured by qRT-PCR. The results were normalized to the expression level of the housekeeping gene GAPDH. The bars show the levels of IL-1β, IL-6 and IL-10 (means ± standard deviations) in hamster peritoneal macrophages. The levels of IL-1β, IL-6 and IL-10 in blank control peritoneal macrophages were assigned a value of 1.0. Different mRNA expression levels between the infected group and the group stimulated with β-glucan and leptospires were compared using one-way ANOVA. *, P < 0.05.

To investigate whether β-glucan prevent hamster from *Leptospira* infection via trained immunity, β-glucan was administered earlier (5 days prior to infection). The mice in the infected control group began to die on the fourth day after challenge, and the 21-day survival rate was 0%. β-Glucan significantly improved the survival rate to 37.5%. There were no deaths in the β-glucan control group (Fig 3A).

The liver, kidney, and lung lesion grades were lower in hamsters coinjected with β-glucan and leptospires than in the infected controls (Fig 3B). Representative images of hamster livers, kidneys and lungs were selected from the group coinjected with β-glucan and leptospires and from the infected controls. The livers of infected control hamsters showed more inflammatory foci and a wider intercellular space than did those of hamsters coinjected with β-glucan and leptospires (Fig 3Ca and 3Cb). Dramatic lesions with hemorrhage were found in renal tissues of the infected controls (Fig 3Cc). In contrast, the kidneys of hamsters coinjected with β-glucan and leptospires showed some evidence of hemorrhage (Fig 3Cd). Severe pulmonary hemorrhages were found in the lungs of infected controls, whereas few hemorrhagic foci were found in hamsters coinjected with β-glucan and leptospires (Fig 3Ce and 3Cf).

To detect the induction of selected genes related to trained immunity, peritoneal macrophages derived from hamsters were stimulated with β-glucan prior to *Leptospira* infection *in vitro*. Administration of β-glucan before *leptospira* challenge led to higher expression of inflammatory cytokines, including IL-1β and IL-6, than *Leptospira* infection alone (Fig 3D). In contrast, the mRNA expression of IL-10 was significantly inhibited by β-glucan compared to that in the control group, which led to even more pronounced suppression of IL-10 expression in the presence of leptospires (Fig 3D). It proved β-glucan had a positive pro-inflammatory response.

## Discussion

In nature, pathogenic *Leptospira* species affect a variety of animal hosts, causing leptospirosis, a reemerging infectious disease [34]. Antibiotics, an effective treatment method for leptospirosis [2], cause toxicity and side effects, and drug resistance and double infections can occur [5]. Therefore, the development of alternatives to antibiotics has become an inevitable requirement in this emerging situation. β-Glucan is a potential immunostimulant. Many studies demonstrate that β-glucan can protect against bacterial and various other infections [35,36] by stimulating immune functions both *in vitro* and *in vivo* [37,38]. However, it is not clear whether β-glucan has immunomodulatory effects on leptospirosis. In the present study, we selected the hamster/*Leptospira* model, which is a suitable model due to its sensitivity to *in vivo* challenge with *Leptospira* [39,40]. Our results showed that β-glucan had preventive potential to leptospirosis in hamsters. Peritoneal macrophages of β-glucan-treated hamsters have increased levels of TLR2, interleukin-1β, interleukin-6 and IFN-γ, indicating that β-glucan has immunostimulatory activity in hamsters.

Golden Syrian hamsters are highly susceptible to *Leptospira* infection and experience severe disease, including a pathological cytokine storm. Mice, on the other hand, are generally resistant to infection [41]. The reason for the difference is the early regulation of proinflammatory mediators in mouse tissues, in contrast with their delayed and massive overexpression in hamster tissues [41]. Some pathogens disrupt the delicate balance of a suitable inflammatory response, tipping it from beneficial to destructive by causing large amounts of positive feedback in immune cells and upregulation of proinflammatory markers—in particular, the cytokines TNF-α, IL-1β, IL-8, and IL-6 [42]. Our results indicated that β-glucan activated IL-1β early cytokine storm.

Emerging evidence has proven that TLR2 and TLR4 play a crucial role in the development of leptospirosis [7,23]. Our findings demonstrated that β-glucan activated TLR2 early but did not significantly alter TLR4 mRNA expression at 2 dpi. A previous study reported that TLR2 activation during early *Leptospira* infection protected against leptospirosis in hamsters [7]. Therefore, β-glucan may exert protective effects against leptospirosis by activating TLR2. Future studies are needed to substantiate this hypothesis. Interestingly, it was found that the burden of *Leptospira* in the lungs was increased, with the inverse pattern in the liver and kidneys. However, the pathological damage to the lung was ameliorated, and the expression of TLR2 in the lungs was inhibited. These results are consistent with the results of previous studies [43].

Moreover, nitric oxide (NO) is an antimicrobial compound produced by inducible nitric oxide synthase (iNOS) in macrophages and endothelial cells, and NO that is excessively formed by iNOS intensifies the inflammatory reaction and causes damage to tissues [27]. Our data showed that β-glucan increased the expression of iNOS in advance and then decreased it at 5 dpi in Kidney, because in the lungs the Lepto by itself had a stronger effect. We speculate that β-glucan enhanced early resistance to *Leptospira* and then protected the body from damage caused by excessive NO.

Trained immunity implies a nonspecific memory of the innate immune response that results in a more robust host response to a second stimulus. Often, trained immunity is described as the memory of the innate immune system [22,44]. In fact, trained immunity phenotypes that share traits with the β-glucan-trained phenotype, such as that generated by the BCG vaccine, seem to have long-lasting effects in humans [15,45]. B-Glucan-induced trained immunity relies on epigenetic changes [17,19]. Our results indicated that, as a result of 5 days of pretreatment with β-glucan, the resistance of hamsters to *Leptospira* was enhanced. The expression of proinflammatory cytokines (IL-1β and IL-6) in hamster peritoneal macrophages was increased at 2 dpi. However, the expression of an anti-inflammatory cytokine (IL-10) was reduced. It proved β-glucan had a positive pro-inflammatory response. We hypothesized that β-glucan exerts its effects by training the immune system. β-Glucan can induce immune responses such as “trained” the host immune system to “recognize” the pathogen or the pro-inflammatory response was trigger prior to challenge to lower the mortality.

Taken together, these results pose an interesting question about the preventable effect and mechanism of β-glucan in hamster/*Leptospira* models. β-Glucan improved survival, alleviated the pathology of leptospirosis, and decreased the abundance of leptospires in hamsters. Based on our findings, we can speculate that β-glucan treatment in the hamster/*Leptospira* model may induce advance upregulation of innate immune signaling pathways or trained immunity, resulting in prevent *Leptospira*. These findings will contribute to a better understanding of the pathogenic mechanism of leptospirosis and reveal new treatment strategies. Additionally, we confirmed that β-glucan could be a treatment agent or vaccine adjuvant for *Leptospira* infection.

## Supporting information

S1 Fig Effect of β-glucan to inflammatory response *in vivo*. The experimental group was injected with β-glucan (10 mg/ml) for 24 h. The TLR2, TLR4, IL-1β and iNOS mRNA levels in the kidneys, livers, and lungs of hamsters were quantified by RT-qPCR. The results were normalized to the expression level of the housekeeping gene GAPDH.
(TIF)
