# Supplementary figures and images for: The preventable efficacy of β-glucan against leptospirosis
Source: PLoS Negl Trop Dis. 2019 Nov 1;13(11):e0007789. doi: 10.1371/journal.pntd.0007789 (PMC6860453; doi:10.1371/journal.pntd.0007789)

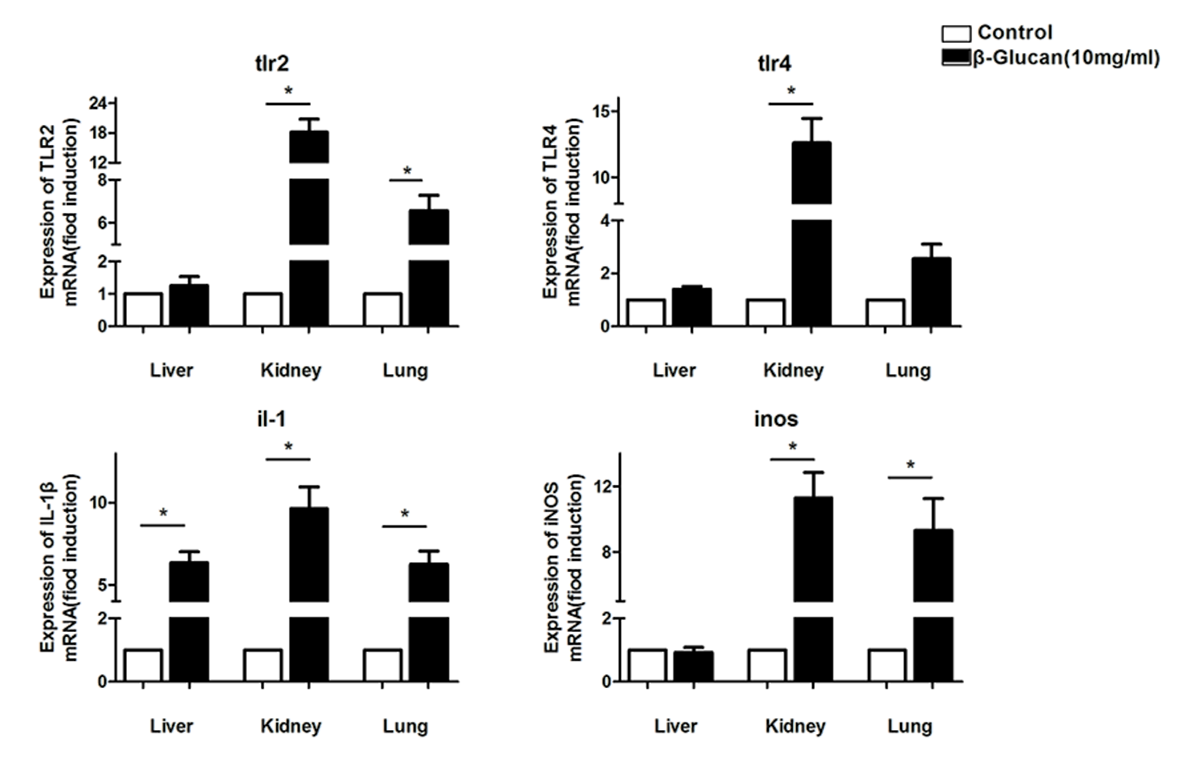

Supplement: S1 Fig — The experimental group was injected with β-glucan (10 mg/ml) for 24 h. The TLR2, TLR4, IL-1β and iNOS mRNA levels in the kidneys, livers, and lungs of hamsters were quantified by RT-qPCR. The results were normalized to the expression level of the housekeeping gene GAPDH. (TIF) [file pntd.0007789.s001.tif]
